# Characterization of tricyclic anti-depressant drugs efficacy via topological indices

**DOI:** 10.1038/s41598-025-05045-6

**Published:** 2025-07-02

**Authors:** Simran Kour, J. Ravi Sankar

**Affiliations:** Department of Mathematics, School of Advanced Sciences, Vellore Institute of Technology, Vellore, Tamil Nadu 632 014 India

**Keywords:** Tricyclic anti-depressant drugs, QSPR analysis, Distance-based topological indices, Python programming, Applied mathematics, Molecular medicine

## Abstract

Within the context of graph theory, a topological index serves as a numerical descriptor that encapsulates the physicochemical properties of a chemical graph. These are particularly useful in cheminformatics, where they serve as a compact representation of the molecule’s structure, capturing various physicochemical properties such as molecular size, shape, branching, and connectivity. These studies are pivotal in the initial phases of drug development, facilitating the identification and optimization of potential pharmaceutical drugs. In this paper, we discuss a range of distance-based topological indices applied to a selection of tricyclic anti-depressant drugs aiming to understand their physicochemical characteristics. Additionally, the quantitative structure–property relationship (QSPR) analysis is explored for distance-based topological indices aim to predict how changes in chemical structure might influence the efficacy and potency of these drugs.

## Introduction

Depression is characterized by the existence of feelings of melancholy, emptiness, or agitation, accompanied with physiological and cognitive changes lasting at least 2 weeks that severely limit the individual’s ability to operate. One of the major effects of depression is related to the loss of memory ability, that is, sometimes they are not able to recall the subject matter and appear to daydream frequently. The effect of memory endurance is often due to pressures from school, home, work, and unfinished tasks. This can lead to a weakening of the memory system, particularly short-term memory^1^. Depression recurrence in young individuals, the onset of other mental diseases, and long-term impairments in interpersonal, social, educational, and occupational functioning are all negative effects of depression. Preventing and addressing depression in young people is crucial, with early intervention targeting predisposing factors, antecedents, and symptoms. High-risk groups encompass individuals with a family history of depression, those exposed to social stressors such as individuals experiencing significant life events, bullying or tumultuous relationships, and certain sub populations, including the ones going through chronic physicochemical health conditions or members of sexual minorities. Clinical precursors commonly involve anxiety, irritability, and depressive symptoms^2^. In contemporary society, depression permeates society, ensnaring individuals irrespective of background. Its insidious grip often manifests in diminished fortitude, heightened irritability, and a loss of verbal expression. While the natural design of our bodies facilitates a gradual return to equilibrium post-shock, humans frequently falter in maintaining resilience against chronic stressors. In the modern milieu, the interplay of financial strains, occupational pressures, and familial discord fosters a breeding ground for anxiety disorders, perpetuating profound emotional and psychological disarray over the long term^3^. Due to the heterogeneity of depression, a step-wise treatment approach is recommended, starting with brief psycho-social interventions, progressing to specific psychological therapy, and potentially leading to anti-depressant medication if needed. To further address the nature of anti-depressant drugs, it is crucial to understand its molecular topology.

Chemical graph theory is a branch of mathematical chemistry that studies how chemical systems can be represented by graphs called chemical graphs. A chemical compound is conventionally depicted as a graph, where its constituent elements are depicted as vertices, while the interlinking bonds are illustrated as edges. By utilizing chemical graphs, topological indices are calculated numerical descriptors formulated to thoroughly describe the chemical system. They are extensively utilized in researching the physicochemical properties of various pharmaceuticals. The Wiener Index, a pioneering concept introduced by Wiener in 1947, stands out as one of the earliest and most extensively utilized topological index. It was initially employed to ascertain the physical attributes of paraffin^4^. It is computed by summing the distances between all pairs of vertices in the molecular graph. Over time, a multitude of topological indices have emerged, such as the Randić index, Balaban index, Zagreb indices, Detour index, Harary index, and molecular connectivity indices, each of these indices highlights unique structural aspects and serves various applications across chemistry and related disciplines^5–7^. In recent years, there has been a significant interest in the use of topological indices in Quantitative Structure–Property Relationships (QSPR) and Quantitative Structure-Activity Relationships (QSAR) investigations^4,8–10^. This model does not require any lab equipment to carry out the analysis which saves time and money^11,12^.

Research has been conducted to explore the use of chemical graph theory and QSPR models in predicting the effects of drugs. Abdul Hakeem et al. evaluated six degree-based topological indices for modeling and characterizing seven heart attack drugs structures to predict physicochemical properties, such as molecular weight and melting point, without extensive lab work, aiding in drug design and optimization^13^. Micheal Arockiaraj et al. developed QSPR models using distance-based topological indices to improve anti-tuberculosis drug design by linking molecular structure to physicochemical properties^14^. Deepa Balasubramaniyan et al. investigated QSPR analysis using sixteen distance-based topological indices to predict six properties of nineteen anti-asthmatic drugs, comparing the results with those from degree-based indices to identify the best predictors and explore new directions^15^. Saima Parveen et al. explored the utility of degree-based topological indices in predicting physicochemical properties of drugs used to treat vitiligo, a skin pigmentation disorder. By analyzing drugs like azathioprine and clobetasol, the study aims to establish mathematical relationships between properties such as polarity and enthalpy and various molecular descriptors. The resulting QSPR model aids in predicting physicochemical properties, potentially informing the design of novel vitiligo treatments and other drugs^16^. Furthermore, researchers have explored the application of topological indices into algebraic theory, illustrating the widening scope and impact of these mathematical methodologies in several scientific disciplines. Rayer *et al.* computed the graph energy and Wiener index of the zero divisor graph for commutative rings by examining the adjacency matrix and calculating the distances between vertices^17^. Furthermore, he investigated the concept of zero-divisor graphs in commutative rings and explored various topological indices offering symmetric physical structures of finite commutative rings^18^. These effects underscore the continuous advancement and adaptability of chemical graph theory in pharmaceutical research and development.

In our study, we focused on tricyclic anti-depressant drugs, representing their molecular structures as hydrogen-depleted chemical graphs. The topological indices mentioned above were further developed and calculated specifically for these drugs. Using distance-based topological indices, we analyzed the structural and functional aspects of these compounds, correlating these indices with their physicochemical properties. The study aimed to evaluate the topological indices of fifteen tricyclic anti-depressant drugs, employ regression models to establish QSPR based on their physicochemical properties, and compare the predicted values with actual properties to validate the reliability of the models. This works builds on our previous research involving anti-cancer drugs, where we applied a similar graph-theoretical approach to derive topological descriptors and study their correlation with molecular properties, thus demonstrating the effectiveness of this methodology across different therapeutic classes^19^.

This article is organized into several sections to provide a comprehensive understanding of the projected work in this research study. “Materials and methods” section describes the materials and methods that are used for calculating the topological indices. In “Main results” section , regression analysis is performed, presenting a comparison between the correlation coefficients and the physicochemical properties, along with a discussion of other statistical parameters. “Discussion” section highlights the implications of the correlation analysis, drawing key insights for tricyclic anti-depressant drugs. Finally, “Conclusion” section presents concluding remarks, discusses the study’s implications, limitations, and outlines potential directions for future research.

## Materials and methods

**Fig. 1:**
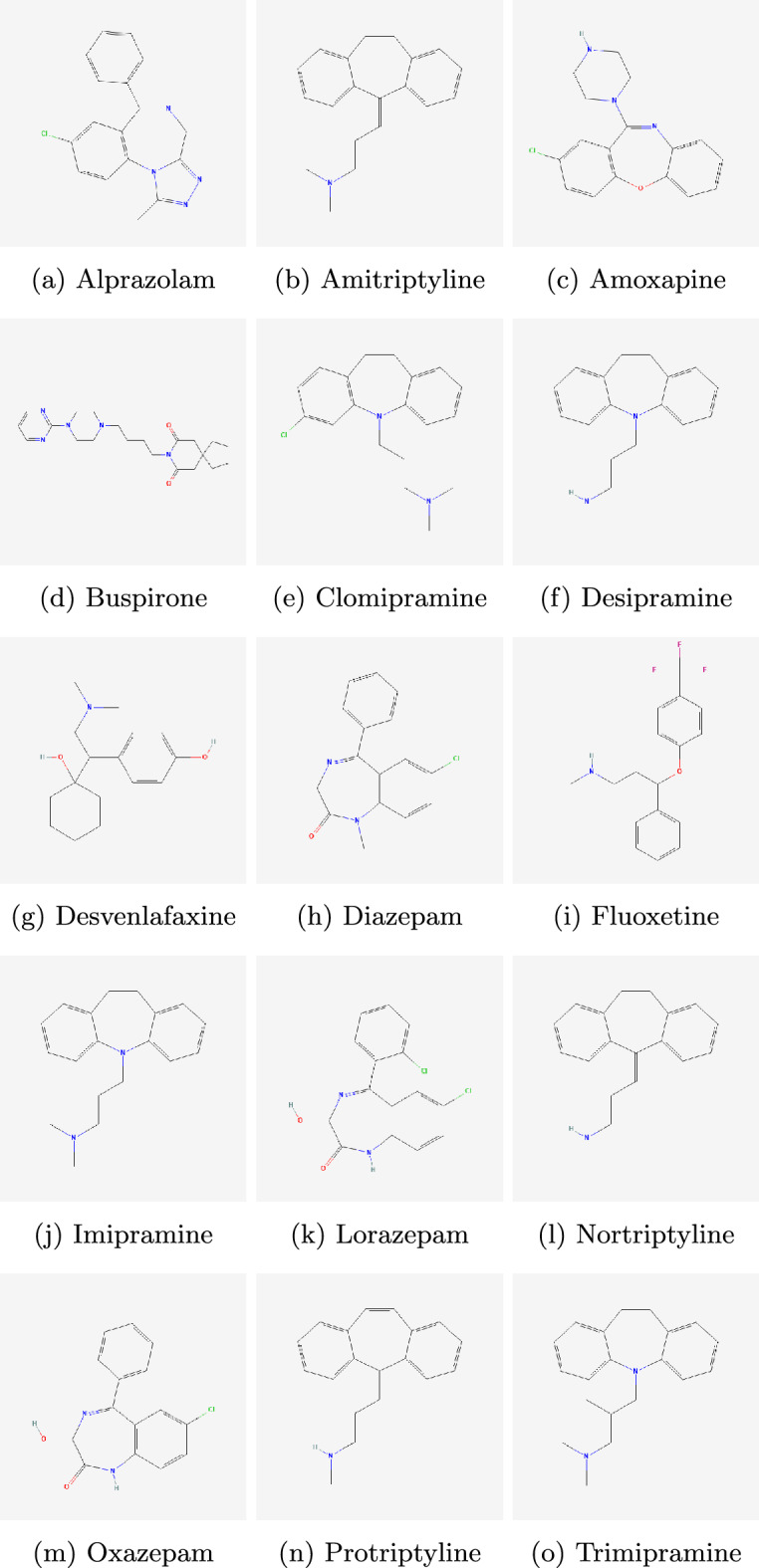
Molecular structures of tricyclic anti-depressant drugs.

In this study, 15 tricyclic anti-depressant drugs were selected for the analysis. The physicochemical properties of these drugs were retrieved from the ChemSpider database^20^. The detailed list of these drugs along with their physicochemical properties is provided in Table 1. Additionally, the chemical structures of these drugs, also retrieved from ChemSpider, are presented in Fig. 1.

**Table 1:** Tricyclic anti-depressant drugs with their physicochemical properties.

Drugs	Boiling point	Melting point	Enthalpy	Flash point	Molar refractivity	Polarizability	Surface tension	Molar volume
Alprazolam	509	228.25	77.9	261.6	88.2	35	52.2	225.6
Alprazolam	509	228.25	77.9	261.6	88.2	35	52.2	225.6
Amitriptyline	398.2	196	64.9	174	91.5	36.3	47	257.8
Amoxapine	469.9	175.5	73.2	238	86.8	34.4	52.1	228.2
Buspirone	613.9	202	91.1	325.1	106.8	42.4	62.4	310.7
Clomipramine	434.2	189.5	69	216.4	93.8	37.2	41.7	281.2
Desipramine	407.4	25	65.9	160.5	84.2	33.4	40	254.3
Desvenlafaxine	403.8	208	69.1	193.2	77.8	30.9	47.9	236.1
Diazepam	497.4	125	76.5	254.6	80.9	32.1	46.1	225.9
Fluoxetine	395.1	180.5	64.5	192.8	79.9	31.7	33	266.7
Imipramine	403.1	174.5	65.4	179.7	88.9	35.3	40.1	269.2
Lorazepam	543.6	167	86.5	282.6	81	32.1	56	211.2
Nortriptyline	403.4	214	65.5	194.9	86.8	34.4	47.3	242.9
Oxazepam	516.6	205.5	83	266.2	76.4	30.3	54.6	201.9
Protriptyline	407.7	170	66	198.3	84.8	33.6	41.2	256.5
Trimipramine	411.8	45	66.4	183.3	93.5	37.1	39.1	286.1

### Topological indices

In chemical graph theory, chemical graphs are a common way to represent pharmaceutical drugs. In these graphs, each vertex represents an atom, and each edge represents the relationship between two atoms. Assume that the collection of edges and vertices that constitutes a molecular graph, expressed through the representation *G*(*V*, *E*). The graphs under study are underlined simple graphs which means there exist no loops and they do not any generate cycles. Distance-based topological indices used are given as follows:

#### Definition 1

The Wiener Index^11^ of the molecular graph G is defined as

1 $$\begin{aligned} W(G)=\sum _{1\le a<b\le n} d(v_a,v_b), \end{aligned}$$

where, $d(v_a,v_b)$ be the distance between the vertices $v_a$ and $v_b$.

#### Definition 2

The Hyper-Wiener Index^12^ of the molecular graph G is defined as

2 $$\begin{aligned} WW(G)=\sum _{1\le a<b\le n}\frac{d(v_a,v_b)+d^2(v_a,v_b)}{2}. \end{aligned}$$

#### Definition 3

The Harary Index^21^ of the molecular graph G is defined as

3 $$\begin{aligned} H(G)=\sum _{1\le a<b\le n} \frac{1}{d(v_a,v_b)}. \end{aligned}$$

#### Definition 4

The Detour Index^22^ of the molecular graph G is defined as

4 $$\begin{aligned} D(G)=\sum _{1\le a<b\le n} D(v_a,v_b), \end{aligned}$$

where, $D(v_a,v_b)$ be the length of the longest path distance between the vertices $v_a$ and $v_b$.

#### Definition 5

The Detour Harary Index^23^ of the molecular graph G is defined as

5 $$\begin{aligned} DH(G)= \sum _{1\le a<b\le n} \frac{1}{D(v_a,v_b)}, \end{aligned}$$

where, $D(v_a,v_b)$ be the length of the longest path distance between the vertices $v_a$ and $v_b$.

The topological indices pertaining to tricyclic anti-depressant drugs are systematically computed and displayed in Table 2. Using the aforementioned formulas, a Python-based algorithm was developed to calculate these indices efficiently. Since distance-based topological indices involve complex computations that are challenging and prone to error when performed manually, Python’s libraries like RDKit and NetworkX were employed. RDKit provided specialized tools for handling chemical data, including the computation of molecular descriptors, while NetworkX facilitated graph-based computations. These libraries significantly enhanced the efficiency, accuracy, and reproducibility of the topological index calculations for the studied compounds. The workflow of the algorithm used for these calculations is also provided in Algorithm 1.

**Algorithm 1:**
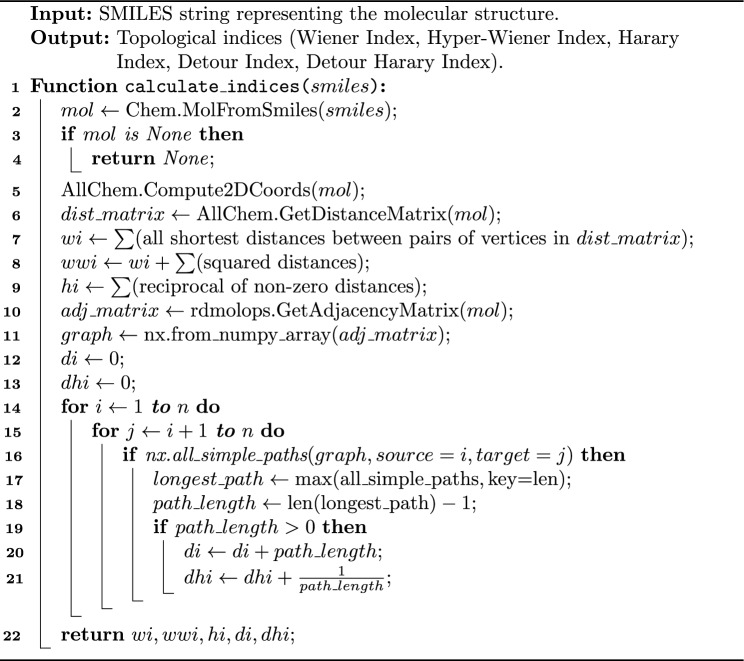
Calculation of topological indices for a molecular structure.

**Table 2:** Topological indices of the tricyclic anti-depressant drugs.

Drugs	Wiener index	Hyper-Wiener index	Harary index	Detour index	Detour Harary index
Alprazolam	926	2770	81.4698	2845	24.1903
Alprazolam	882	2780	70.1254	2581	26.2091
Amoxapine	936	2813	80.7849	3005	22.4103
Buspirone	2514	13,028	102.7360	3764	57.3298
Clomipramine	995	3194	78.0429	2861	28.6732
Desipramine	759	2282	68.0484	2328	22.5787
Desvenlafaxine	672	1979	61.8337	1056	38.8190
Diazepam	726	2077	69.4905	1901	24.9069
Fluoxetine	1148	4312	72.9427	1604	49.6266
Imipramine	882	2780	70.1256	2581	26.2091
Lorazepam	819	2365	74.9607	2148	27.8257
Nortriptyline	759	2282	68.0484	2328	22.5787
Oxazepam	727	2076	69.3488	1909	24.8700
Protriptyline	759	2282	68.0484	2328	22.5787
Trimipramine	979	3081	78.4616	2808	30.3429

## Main results

### Regression analysis

This work analyses the drug’s computable structure concerning five topological indices in order to model the tenacity of QSPR. The eight physicochemical characteristics of the fifteen drugs listed in Table 1 are utilized in the management of depressant drugs. The ensuing equation is utilized to establish correlations between distinct physicochemical properties of tricyclic anti-depressant drugs used for the treatment of depression with pertinent topological indices. The table data for topological indices and physicochemical properties shows normally distributed values. The following linear regression model has been considered:

6 $$\begin{aligned} P=A+b (TI). \end{aligned}$$

Within this context, P denotes the physicochemical property of the provided drugs, TI represents the topological index, A serves as a constant term, and b stands for the regression coefficient. The constants A and regression coefficients b are computed via SPSS software, leveraging data encompassing eight physicochemical properties and five distance-based topological indices across fifteen drug molecules. Employing Eq. (6), the ensuing linear regression models encapsulate the prescribed distance-based topological indices.

1. Regression models for Wiener index

**Table:** 

Boiling point	$= 368.8107 + 0.0886[W(G)]$
Melting point	$= 147.1065 + 0.0207[W(G)]$
Enthalpy	$= 62.7397 + 0.0099[W(G)]$
Flash point	$= 164.9316 + 0.0585[W(G)]$
Molar refractivity	$= 73.7389 + 0.0135[W(G)]$
Polarizability	$= 29.2304 + 0.0054[W(G)]$
Surface tension	$= 39.588 + 0.0074[W(G)]$
Molar volume	$= 207.4866 + 0.0443[W(G)]$

2. Regression models for Hyper-Wiener index

**Table:** 

Boiling point	$= 406.0619 + 0.0145[WW(G)]$
Melting point	$= 155.4408 + 0.0035[WW(G)]$
Enthalpy	$= 66.8414 + 0.0016[WW(G)]$
Flash point	$= 189.6595 + 0.0095[WW(G)]$
Molar refractivity	$= 79.6897 + 0.0021[WW(G)]$
Polarizability	$= 31.5994 + 0.0008[WW(G)]$
Surface tension	$= 42.5531 + 0.0012[WW(G)]$
Molar volume	$= 226.7030 + 0.0071[WW(G)]$

3. Regression models for Harary index

**Table:** 

Boiling point	$= 86.9197 + 4.9452[H(G)]$
Melting point	$= 111.1755 + 0.752[H(G)]$
Enthalpy	$= 31.3427 + 0.5516[H(G)]$
Flash point	$= -24.9064 + 3.3153[H(G)]$
Molar refractivity	$= 38.9334 + 0.6436[H(G)]$
Polarizability	$= 15.407 + 0.2558[H(G)]$
Surface tension	$= 15.4636 + 0.4206[H(G)]$
Molar volume	$= 134.0205 + 1.5644[H(G)]$

4. Regression models for Detour index

**Table:** 

Boiling point	$= 349.5813 + 0.0440[D(G)]$
Melting point	$= 171.0697 - 0.0017[D(G)]$
Enthalpy	$= 62.8632 + 0.0040[D(G)]$
Flash point	$= 160.0726 + 0.0256[D(G)]$
Molar refractivity	$= 61.1895 + 0.0106[D(G)]$
Polarizability	$= 24.2739 + 0.0042[D(G)]$
Surface tension	$= 36.5795 + 0.0042[D(G)]$
Molar volume	$= 191.7104 + 0.0244[D(G)]$

5. Regression models for Detour Harary index

**Table:** 

Boiling point	$= 395.0783 + 1.9791[DH(G)]$
Melting point	$= 135.2215 + 1.063[DH(G)]$
Enthalpy	$= 64.8647 + 0.2492[DH(G)]$
Flash point	$= 177.4057 + 1.4697[DH(G)]$
Molar refractivity	$= 78.6413 + 0.2709[DH(G)]$
Polarizability	$= 31.1569 + 0.1088[DH(G)]$
Surface tension	$= 43.7431 + 0.0992[DH(G)]$
Molar volume	$= 202.7603 + 1.5872[DH(G)]$

### Comparison between correlation coefficient and physicochemical properties of the drugs

**Fig. 2:**
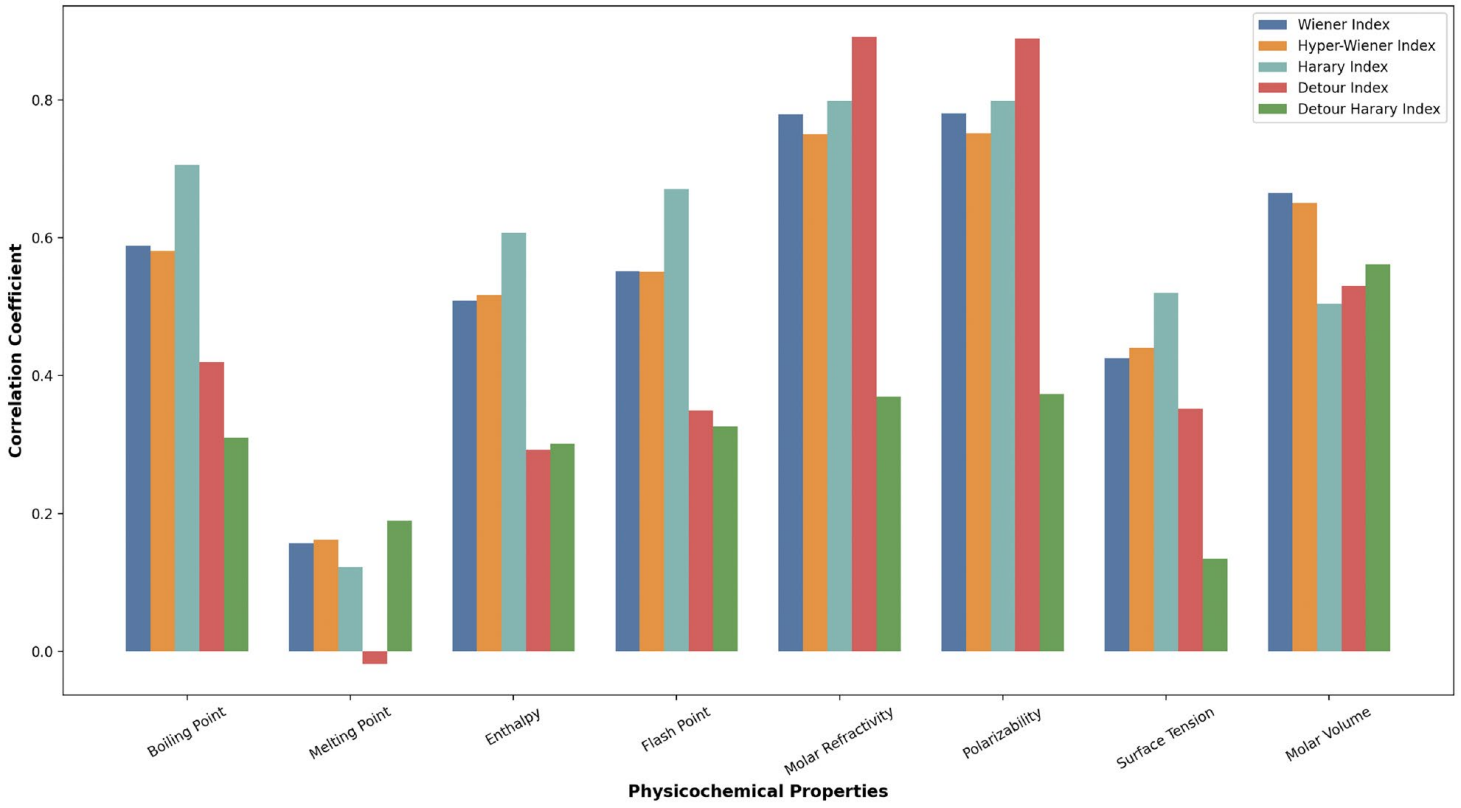
Correlation between topological indices and physicochemical properties.

Within Table 3, the computed correlation coefficients delineating the relationship between eight physicochemical properties and the topological indices of tricyclic anti-depressant drugs have presented and strong correlations are highlighted in bold. These correlation coefficient were calculated to quantify the strength of relationship between molecular properties and topological indices. In addition to the correlation coefficients presented in Table 3, Fig. 2 provides a bar graph illustrating the connections between topological indices and physicochemical properties of tricyclic anti-depressant drugs.

**Table 3:** Correlation coefficient of physicochemical properties.

Topological indices	Boiling point	Melting point	Enthalpy	Flash point	Molar refractivity	Polarizability	Surface tension	Molar volume
Wiener index	0.5884	0.1564	0.5089	0.5507	**0.7788**	**0.7802**	0.4244	0.6648
Hyper-Wiener index	0.5805	0.1615	0.5167	0.5499	**0.7501**	**0.7517**	0.4397	0.6501
Harary index	**0.7055**	0.1223	0.6071	0.6709	**0.7987**	**0.7985**	0.5195	0.504
Detour index	0.4197	− 0.0183	0.2925	0.3486	**0.8908**	**0.8888**	0.3515	0.5298
Detour Harary index	0.3098	0.1896	0.301	0.3263	0.3689	0.3725	0.1344	0.5609

Significant values are in bold.

### Computation of statistical parameters and standard error of the estimate

The statistical parameters are examined for all the considered topological indices and physicochemical properties are shown in Tables 4, 5, 6, 7 and 8, provides a comprehensive understanding of the relationships within the dataset. Parameters such as the number of drugs considered (*N*), constant (*A*), regression coefficient (*b*), correlation coefficient ($R^2$), Fisher’s statistic (*F*), and significance value (*p*) offer insights into the strength and significance of associations observed. For interpretation, a p-value less than 0.05 was considered statistically significant, whereas a p-value greater than 0.05 indicated a lack of statistical significance. These metrics not only quantify the relationships but also facilitate comparisons and informed interpretations. Table 9 depicts the statistical analysis of estimation error for the physicochemical properties of the tricyclic anti-depressant drugs. This metric highlights the accuracy and reliability of the predictive models applied for these properties. Evaluation of the statistical analysis of estimation error provides insight into the precision of predictions, thereby fortifying confidence and reliability in the resulting conclusions of the statistical analyses undertaken.

**Table 4:** Statistical measures incorporated in the QSPR modeling framework for the Wiener Index.

Physicochemical property	*N*	*A*	*b*	$\varvec{R^2}$	*F*	*p*	Indicator
Boiling point	15	368.8107	0.0886	0.3463	6.8854	0.0210	Significant
Melting point	15	147.1065	0.0207	0.0245	0.3258	0.5779	Not significant
Enthalpy	15	62.7397	0.0099	0.2589	4.5426	0.0527	Not significant
Flash point	15	164.9316	0.0585	0.3033	5.6585	0.0334	Significant
Molar refractivity	15	73.7389	0.0135	0.6065	20.0359	0.0006	Significant
Polarizability	15	29.2304	0.0054	0.6087	20.2193	0.0006	Significant
Surface tension	15	39.588	0.0074	0.1801	2.8555	0.1149	Not significant
Molar volume	15	207.4866	0.0443	0.4419	10.2935	0.0069	Significant

**Table 5:** Statistical measures incorporated in the QSPR modeling framework for the Hyper-Wiener Index.

Physiochemical property	*N*	*A*	*b*	$\varvec{R^2}$	*F*	*p*	Indicator
Boiling point	15	406.0619	0.0145	0.3475	6.9228	0.0207	Significant
Melting point	15	155.4408	0.0035	0.0261	0.3483	0.5652	Not significant
Enthalpy	15	66.8414	0.0016	0.267	4.7354	0.0486	Significant
Flash point	15	189.6595	0.0095	0.3024	5.6364	0.0337	Significant
Molar refractivity	15	79.6897	0.0021	0.5627	16.7291	0.0013	Significant
Polarizability	15	31.5994	0.0008	0.5651	16.8912	0.0012	Significant
Surface tension	15	42.5531	0.0012	0.1934	3.1166	0.1101	Not significant
Molar volume	15	226.7030	0.0071	0.4226	9.5147	0.0087	Significant

**Table 6:** Statistical measures incorporated in the QSPR modeling framework for the Harary index.

Physiochemical property	*N*	*A*	*b*	$\varvec{R^2}$	*F*	*p*	Indicator
Boiling point	15	86.9197	4.9452	0.4978	12.8851	0.0033	Significant
Melting point	15	111.1755	0.752	0.0149	0.1973	0.6642	Not significant
Enthalpy	15	31.3427	0.5516	0.3686	7.5885	0.0164	Significant
Flash point	15	− 24.9064	3.3153	0.4501	10.6414	0.0062	Significant
Molar refractivity	15	38.9334	0.6436	0.6379	22.8992	0.0004	Significant
Polarizability	15	15.407	0.2558	0.6376	22.8751	0.0004	Significant
Surface tension	15	15.4636	0.4206	0.2699	4.8048	0.0472	Significant
Molar volume	15	134.0205	1.5649	0.254	4.4271	0.0554	Not significant

**Table 7:** Statistical measures incorporated in the QSPR modeling framework for the Detour index.

Physiochemical property	*N*	*A*	*b*	$\varvec{R^2}$	*F*	*p*	Indicator
Boiling point	15	349.5813	0.0440	0.1761	2.7793	0.1194	Not significant
Melting point	15	171.0697	− 0.0017	0.0003	0.0044	0.9482	Not significant
Enthalpy	15	62.8632	0.0040	0.0856	1.2163	0.2901	Not significant
Flash point	15	160.0726	0.0256	0.1215	1.798	0.2029	Not significant
Molar refractivity	15	61.1895	0.0106	0.7935	49.9422	0.0000	Significant
Polarizability	15	24.2739	0.0042	0.7899	49.8686	0.0000	Significant
Surface tension	15	36.5795	0.0042	0.1235	1.8321	1.1989	Not significant
Molar volume	15	191.7104	0.0244	0.2807	5.0723	0.0422	Significant

**Table 8:** Statistical measures incorporated in the QSPR modeling framework for the Detour Harary index.

Physiochemical property	*N*	*A*	*b*	$\varvec{R^2}$	*F*	*p*	Indicator
Boiling point	15	395.0783	1.9971	0.0960	1.3803	0.2611	Not significant
Melting point	15	135.2215	1.063	0.0360	0.4849	0.4985	Not significant
Enthalpy	15	64.8647	0.2492	0.0906	1.2949	0.2757	Not significant
Flash point	15	177.4057	1.4697	0.1065	1.5494	0.2352	Not significant
Molar refractivity	15	78.6413	0.2709	0.1361	2.0473	0.1761	Not significant
Polarizability	15	31.1569	0.1088	0.1387	2.0942	0.1715	Not significant
Surface tension	15	43.7431	0.0992	0.0181	0.2392	0.6329	Not significant
Molar volume	15	202.7603	1.5872	0.3146	5.9679	0.0296	Significant

**Table 9:** Statistical analysis of estimation error.

Topological indices	Boiling point	Melting point	Enthalpy	Flash point	Molar refractivity	Polarizability	Surface tension	Molar volume
Wiener index	56.4722	60.5322	7.7931	41.1013	5.0374	1.997	7.3054	23.1125
Hyper-Wiener index	56.4192	60.4811	7.7506	41.1257	5.3102	2.1052	7.246	23.5088
Harary index	49.4968	60.8262	7.1936	36.5138	4.8323	1.9216	6.8939	26.7209
Detour index	63.3954	61.2758	8.6569	46.1522	3.6495	1.4633	7.5533	26.2397
Detour Harary index	66.4076	60.174	8.6331	46.5448	7.464	2.9625	7.9947	25.6127

## Discussion

The study examined the relationship between five topological indices and eight physicochemical properties of fifteen tricyclic anti-depressant drugs. The data in Table 3 reveals several patterns, particularly strong correlations ($r>0.7$, highlighted in bold) were observed between most topological indices and two specific physicochemical properties, molar refractivity and polarizability. The Detour Index demonstrated the strongest correlations with these properties ($r=0.8908$ and $r=0.8888$, respectively), suggesting that this topological index may serve as an effective predictor for these molecular characteristics. In contrast all indices showed relatively weak correlations with melting point ($r<0.2$), indicating that these indices have minimal influence on this particular property.

Figure 2 visually represents these correlation patterns, with the bar heights corresponding to the correlation coefficient magnitudes. These graphical representation clearly illustrates the consistently high correlation values for molar refractivity and polarizability across all indices. Additionally, the figure reveals a moderate correlation pattern for boiling point, flash point, and molar volume ($r\approx 0.5-0.7$) for most indices, while surface tension shows variable correlation strengths depending on the specific topological index employed.

The QSPR analysis quantified the identified correlations using key statistical metrics such as $R^2$, the *F*-statistic, *p*-value, and regression coefficients (*A*, *b*) in Tables 4, 5, 6, 7, 8 and 9. Both molar refractivity and polarizability demonstrated strong associations with indices like the Detour Index and Wiener Index. Specifically, polarizability achieved $R^2$ = 0.7899, *F* = 49.8686, *p* = 0.0 with the Detour Index, and $R^2$ = 0.6376, *F* = 22.8751, *p* = 0.0004 with the Harary Index, suggesting that these indices are key for predicting molecular interactions. Likewise, molar refractivity exhibited excellent predictive accuracy, with $R^2$ = 0.7935, *F* = 49.9422, *p* = 0.0 for the Detour Index and $R^2$ = 0.6065, *F* = 20.0359, *p* = 0.0006 for the Wiener Index. These findings underscore the reliability of these indices in modeling molecular properties strongly influenced by atomic arrangements.

In contrast, melting point and surface tension exhibited weak correlations, indicated by low $R^2$ values and non-significant *p*-values. For instance, the Detour Index showed poor performance in predicting melting point ($R^2$ = 0.0003, *p* = 0.9482) and surface tension ($R^2$ = 0.1235, *p* = 1.1989), indicating that these properties are influenced by factors not captured by topological descriptors. Moderate correlations were observed for boiling point and flash point, especially with the Wiener and Hyper-Wiener Indices. Boiling point exhibited $R^2$ = 0.3463, *F* = 6.8854, and *p* = 0.0210 with the Wiener Index, suggesting a moderate dependence on molecular topology. This analysis underscores the value of topological indices like the Wiener Index, Harary Index, and Detour Index for predicting properties such as molar Refractivity and polarizability, which are pivotal in molecular design. However, the weak correlations for melting point and surface tension suggest a need to explore advanced techniques, such as non-linear indices or hybrid modeling approaches, for improved accuracy. The findings underscore the value of tailoring QSPR models to specific physicochemical properties to enhance predictive performance and guide future studies.

## Conclusion

According to QSPR modeling, this study investigates the important relationships between the spatial arrangement of atoms and physicochemical properties in tricyclic anti-depressant drugs through QSPR analysis. It was observed that certain topological indices are effective in predicting properties like molar refractivity and polarizability. These results show that molecular structure plays an important role in determining specific drug characteristics and highlight the usefulness of topological indices in estimating such properties. This approach can support faster drug development by helping identify suitable drug candidates more efficiently and reducing the need for extensive laboratory testing.

### Limitations

On the contrary, there was no apparent correlation detected between distance-based topological indices and the melting point, enthalpy, flash point, surface tension, and molar volume of tricyclic anti-depressant drugs. Moreover, the Detour Harary Index did not provide a good correlation with any of the physicochemical properties. These findings suggest the need for further research and refinement of predictive models to encompass a broader range of physicochemical properties relevant to drug development.

### Future scope

To advance the findings of this study, future investigations could broaden their scope beyond tricyclic anti-depressants to encompass a diverse range of chemical structures and other pharmaceutical compounds. By incorporating a wider array of topological indices and leveraging advanced computational techniques like machine learning can enhance predictive accuracy. Validating these methodologies across diverse drug categories will ensure their broader applicability and relevance. Furthermore, integrating computational predictions with experimental data, as well as forging novel cheminformatics tools, can streamline drug development procedures.

## Supplementary Information

Supplementary Information.

## Figures and Tables

**Fig. 3:**
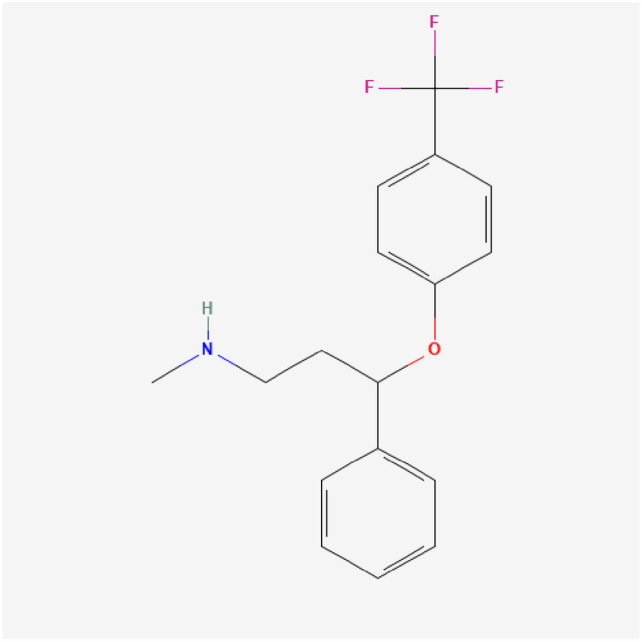
Molecular structure of the Fluoxetine.

**Fig. 4:**
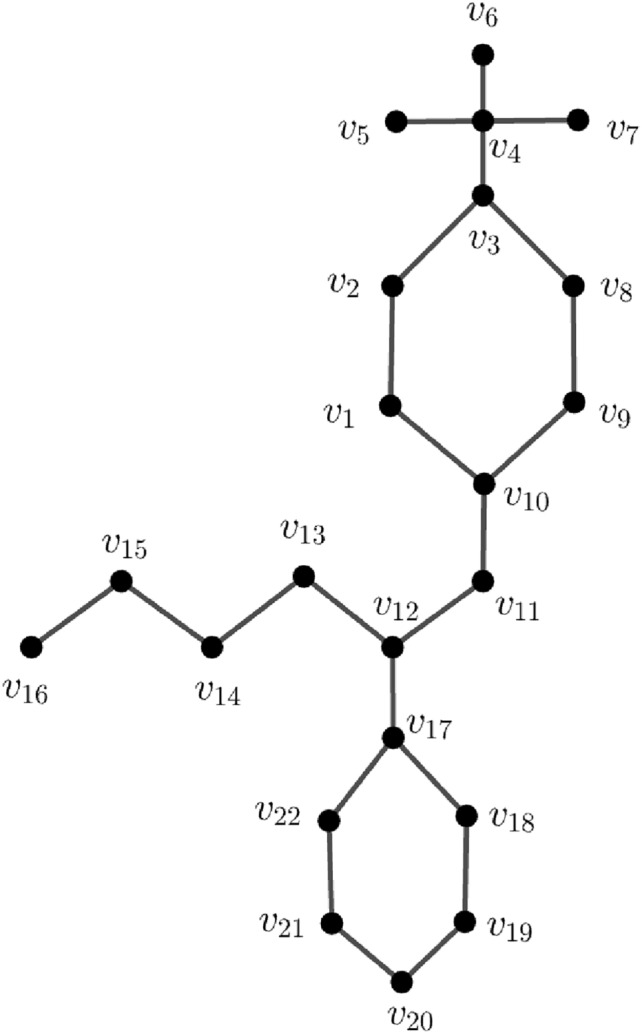
Chemical graph of Fluoxetine.

## Data Availability

All molecular structures, their properties and, their topological indices, used in this study are provided as part of the main manuscript. Scripts and software for data analysis are available at the following repository: https://github.com/simran2410/Topological_Drug_Metrics.git. All necessary files and metadata are included to ensure reproducibility of the study.

## Appendix 1: Detailed calculation of Wiener index

To illustrate the computation of the Wiener index, we provide a step-by-step calculation for Fluoxetine from Table 2. Figure 3 presents the molecular structure of the Fluoxetine, where Fig. 4 shows its chemical graph.

The Wiener index (*W*) is defined as the sum of all shortest-path distances between two vertices in the molecular graph:

$$\begin{aligned} W(G)=\sum _{1\le a<b\le n} d(v_a,v_b), \end{aligned}$$

where $d_{v_a,v_b}$ is the shortest path between vertex $v_a$ and vertex $v_b$.

Consider the distance matrix:

$$\begin{aligned} \begin{array}{c|*{22}{c}} & v_1 & v_2 & v_3 & v_4 & v_5 & v_6 & v_7 & v_8 & v_9 & v_{10} & v_{11} & v_{12} & v_{13} & v_{14} & v_{15} & v_{16} & v_{17} & v_{18} & v_{19} & v_{20} & v_{21} & v_{22} \\ \hline v_1 & 0 & 1 & 2 & 3 & 4 & 4 & 4 & 3 & 2 & 1 & 2 & 3 & 4 & 5 & 6 & 7 & 4 & 5 & 6 & 7 & 6 & 5 \\ v_2 & 1 & 0 & 1 & 2 & 3 & 3 & 3 & 2 & 3 & 2 & 3 & 4 & 5 & 6 & 7 & 8 & 5 & 6 & 7 & 8 & 7 & 6 \\ v_3 & 2 & 1 & 0 & 1 & 2 & 2 & 2 & 1 & 2 & 3 & 4 & 5 & 6 & 7 & 8 & 9 & 6 & 7 & 8 & 9 & 8 & 7 \\ v_4 & 3 & 2 & 1 & 0 & 1 & 1 & 1 & 2 & 3 & 4 & 5 & 6 & 7 & 8 & 9 & 10 & 7 & 8 & 9 & 10 & 9 & 8 \\ v_5 & 4 & 3 & 2 & 1 & 0 & 2 & 2 & 3 & 4 & 5 & 6 & 7 & 8 & 9 & 10 & 11 & 8 & 9 & 10 & 11 & 10 & 9 \\ v_6 & 4 & 3 & 2 & 1 & 2 & 0 & 2 & 3 & 4 & 5 & 6 & 7 & 8 & 9 & 10 & 11 & 8 & 9 & 10 & 11 & 10 & 9 \\ v_7 & 4 & 3 & 2 & 1 & 2 & 2 & 0 & 3 & 4 & 5 & 6 & 7 & 8 & 9 & 10 & 11 & 8 & 9 & 10 & 11 & 10 & 9 \\ v_8 & 3 & 2 & 1 & 2 & 3 & 3 & 3 & 0 & 1 & 2 & 3 & 4 & 5 & 6 & 7 & 8 & 5 & 6 & 7 & 8 & 7 & 6 \\ v_9 & 2 & 3 & 2 & 3 & 4 & 4 & 4 & 1 & 0 & 1 & 2 & 3 & 4 & 5 & 6 & 7 & 4 & 5 & 6 & 7 & 6 & 5 \\ v_{10} & 1 & 2 & 3 & 4 & 5 & 5 & 5 & 2 & 1 & 0 & 1 & 2 & 3 & 4 & 5 & 6 & 3 & 4 & 5 & 6 & 5 & 4 \\ v_{11} & 2 & 3 & 4 & 5 & 6 & 6 & 6 & 3 & 2 & 1 & 0 & 1 & 2 & 3 & 4 & 5 & 2 & 3 & 4 & 5 & 4 & 3 \\ v_{12} & 3 & 4 & 5 & 6 & 7 & 7 & 7 & 4 & 3 & 2 & 1 & 0 & 1 & 2 & 3 & 4 & 1 & 2 & 3 & 4 & 3 & 2 \\ v_{13} & 4 & 5 & 6 & 7 & 8 & 8 & 8 & 5 & 4 & 3 & 2 & 1 & 0 & 1 & 2 & 3 & 2 & 3 & 4 & 5 & 4 & 3 \\ v_{14} & 5 & 6 & 7 & 8 & 9 & 9 & 9 & 6 & 5 & 4 & 3 & 2 & 1 & 0 & 1 & 2 & 3 & 4 & 5 & 6 & 5 & 4 \\ v_{15} & 6 & 7 & 8 & 9 & 10 & 10 & 10 & 7 & 6 & 5 & 4 & 3 & 2 & 1 & 0 & 1 & 4 & 5 & 6 & 7 & 6 & 5 \\ v_{16} & 7 & 8 & 9 & 10 & 11 & 11 & 11 & 8 & 7 & 6 & 5 & 4 & 3 & 2 & 1 & 0 & 5 & 6 & 7 & 8 & 7 & 6 \\ v_{17} & 4 & 5 & 6 & 7 & 8 & 8 & 8 & 5 & 4 & 3 & 2 & 1 & 2 & 3 & 4 & 5 & 0 & 1 & 2 & 3 & 2 & 1 \\ v_{18} & 5 & 6 & 7 & 8 & 9 & 9 & 9 & 6 & 5 & 4 & 3 & 2 & 3 & 4 & 5 & 6 & 1 & 0 & 1 & 2 & 3 & 2 \\ v_{19} & 6 & 7 & 8 & 9 & 10 & 10 & 10 & 7 & 6 & 5 & 4 & 3 & 4 & 5 & 6 & 7 & 2 & 1 & 0 & 1 & 2 & 3 \\ v_{20} & 7 & 8 & 9 & 10 & 11 & 11 & 11 & 8 & 7 & 6 & 5 & 4 & 5 & 6 & 7 & 8 & 3 & 2 & 1 & 0 & 1 & 2 \\ v_{21} & 6 & 7 & 8 & 9 & 10 & 10 & 10 & 7 & 6 & 5 & 4 & 3 & 4 & 5 & 6 & 7 & 2 & 3 & 2 & 1 & 0 & 1 \\ v_{22} & 5 & 6 & 7 & 8 & 9 & 9 & 9 & 6 & 5 & 4 & 3 & 2 & 3 & 4 & 5 & 6 & 1 & 2 & 3 & 2 & 1 & 0 \\ \end{array} \end{aligned}$$

Then:

$$\begin{aligned} \begin{aligned} W&= \sum _{a=1}^{21} d_{v_a,v_b} \\&= 84 + 91 + 97 + 108 + 124 + 122 + 120 + 75 + 61 + 48 \\&\quad + 36 + 25 + 27 + 30 + 34 + 39 + 9 + 8 + 6 + 3 + 1 \\&= 1148 \end{aligned} \end{aligned}$$

Hence, the Wiener index for this compound is $W = 1148$.

The same procedure was followed for the remaining compounds in Table 2 to compute their respective topological indices. This process was uniformly applied to derive all considered indices.
