# Supplementary material for: Characterization of tricyclic anti-depressant drugs efficacy via topological indices
Source: Sci Rep. 2025 Jul 2;15:22853. doi: 10.1038/s41598-025-05045-6 (PMC12217923; doi:10.1038/s41598-025-05045-6)
Supplement: Supplementary file 1 — Supplementary Information. [file 41598_2025_5045_MOESM1_ESM.pdf]

Supplementary Material

Comparison of Actual and Computed Values for Physicochemical Properties from Regression Models

The comparison between actual values of physicochemical properties and the computed values derived from topological indices and the regression models forms a critical aspect of this study. While the values for melting point are obtained are the experimental values, the values obtained for other properties are predicted values. Tables 1 - 8 present a comprehensive overview of these comparisons, allowing for a detailed examination of the accuracy and reliability of the models in predicting various physicochemical properties. This evaluation validates the efficacy of the proposed methodologies and provides insights into their potential applications in drug design and optimization.

**Name of the drug**	**Boiling point (°C at 760 mmHg)**	**Boiling point computed via the regression model with the Wiener Index**	**Boiling point computed via the regression model with the Hyper-Wiener Index**	**Boiling point computed via the regression model with the Harary Index**	**Boiling point computed via the regression model with the Detour Index**	**Boiling point computed via the regression model with the Detour Harary Index**
Alprazolam	509.0±60.0	450.8543	446.2269	489.8042	474.7613	442.9533
Amitriptyline	398.2±21.0	446.9559	446.3719	433.7038	463.1453	446.9487
Amoxapine	469.9±55.0	451.7403	446.8504	486.4172	481.8013	439.4305
Buspirone	613.9±65.0	591.5511	594.9679	594.9698	515.1973	508.5397
Clomipramine	434.2±45.0	456.9677	452.3749	472.8574	475.4653	451.8254
Desipramine	407.4±44.0	436.0581	439.1509	423.4326	452.0133	439.7638
Desvenlafaxine	403.8±25.0	428.3499	434.7574	392.6997	396.0453	471.9050
Diazepam	497.4±45.0	433.1343	436.1784	430.5641	433.2253	444.3715
Fluoxetine	395.1±42.0	470.5235	468.5859	447.6359	420.1573	493.2943
Imipramine	403.1±44.0	446.9559	446.3719	433.7048	444.0933	446.9487
Lorazepam	543.6±60.0	441.3741	440.3544	457.6154	452.0133	450.1481
Nortriptyline	403.4±24.0	436.0581	439.1509	423.4326	433.5773	439.7638
Oxazepam	516.6±60.0	433.2229	436.1639	429.8634	452.0133	444.2985
Protriptyline	407.7±14.0	436.0581	439.1509	423.4326	452.0133	439.7638
Trimipramine	411.8±44.0	455.5501	450.7364	474.9280	473.1333	455.1299

Table 1: Comparing the actual boiling point with those determined computationally through regression modeling

Name of the drug	Melting point (°C)	Melting point computed via the regression model with the Wiener Index	Melting point computed via the regression model with the Hyper-Wiener Index	Melting point computed via the regression model with the Harary Index	Melting point computed via the regression model with the Detour Index	Melting point computed via the regression model with the Detour Harary Index
Alprazolam	228.25	166.2747	165.1358	172.4408	166.2332	160.9358
Amitriptyline	196	165.3639	165.1708	163.9098	166.6820	163.0818
Amoxapine	175.5	166.4817	165.2863	171.9257	165.9612	159.0436
Buspirone	202	199.1463	201.0388	188.4330	164.6709	196.1631
Clomipramine	189.5	167.7030	166.6198	169.8638	166.2060	165.7011
Desipramine	25	162.8178	163.4278	162.3479	167.1121	159.2227
Desvenlafaxine	208	161.0169	162.3673	157.6744	169.2745	176.4861
Diazepam	125	162.1347	162.7103	163.4324	167.8380	161.6975
Fluoxetine	180.5	170.8701	170.5328	166.0284	168.3429	187.9746
Imipramine	174.5	165.3639	165.1708	163.9098	166.6820	163.0818
Lorazepam	167	164.0598	163.7183	167.5459	167.4181	164.8002
Nortriptyline	214	162.8178	163.4278	162.3479	167.1121	159.2227
Oxazepam	205.5	162.1554	162.7068	163.3258	167.8244	161.6583
Protriptyline	170	162.8178	163.4278	162.3479	167.1121	159.2227
Trimipramine	45	167.3718	166.2243	170.1786	166.2961	167.4760

Table 2: Comparing the actual melting point with those determined computationally through regression modeling

**Name of the drug**	**Enthalpy (kJ/mol)**	**Enthalpy computed via the regression model with the Wiener Index**	**Enthalpy computed via the regression model with the Hyper-Wiener Index**	**Enthalpy computed via the regression model with the Harary Index**	**Enthalpy computed via the regression model with the Detour Index**	**Enthalpy computed via the regression model with the Detour Harary Index**
Alprazolam	77.9±3.0	71.9071	71.2734	76.2814	74.2432	70.8929
Amitriptyline	64.9±3.0	71.4715	71.2894	70.0239	73.1872	71.3960
Amoxapine	73.2±3.0	72.0061	71.3422	75.9037	74.8832	70.4493
Buspirone	91.1±3.0	87.6283	87.6862	88.0119	77.9192	79.1513
Clomipramine	69±3.0	72.5902	71.9518	74.3912	74.3072	72.0101
Desipramine	65.9±3.0	70.2538	70.4926	68.8782	72.1752	70.4913
Desvenlafaxine	69.1±3.0	69.3925	70.0078	65.4502	67.0872	74.5384
Diazepam	76.5±3.0	69.9271	70.1646	69.6737	70.4672	71.0715
Fluoxetine	64.5±3.0	74.1049	73.7406	71.5779	69.2792	77.2316
Imipramine	65.4±3.0	71.4715	71.2894	70.0239	73.1872	71.3960
Lorazepam	86.5±3.0	70.8478	70.6254	72.6910	71.4552	71.7989
Nortriptyline	65.5±3.0	70.2538	70.4926	68.8782	72.1752	70.4913
Oxazepam	83.0±3.0	69.9370	70.1646	69.5955	70.4992	71.0623
Protriptyline	66.0±3.0	70.2538	70.4926	68.8782	72.1752	70.4913
Trimipramine	66.4±3.0	72.4318	71.7710	74.6221	74.0952	72.4262

Table 3: Comparing the enthalpy with those determined computationally through regression modeling

**Name of the drug**	**Flash point (°C)**	**Flash point computed via the regression model with the Wiener Index**	**Flash point computed via the regression model with the Hyper-Wiener Index**	**Flash point computed via the regression model with the Harary Index**	**Flash point computed via the regression model with the Detour Index**	**Flash point computed via the regression model with the Detour Harary Index**
Alprazolam	261.6±32.9	219.1026	215.9745	245.1904	232.9046	212.9575
Amitriptyline	174.0±18.9	216.5286	216.0695	207.5803	226.1462	215.9245
Amoxapine	238.0±31.5	219.6876	216.3830	242.9198	237.0006	210.3414
Buspirone	325.1±34.3	312.0006	313.4255	315.6943	256.4310	261.6626
Clomipramine	216.4±28.7	223.1391	220.0025	233.8292	233.3142	219.5460
Desipramine	160.5±19.4	209.3331	211.3385	200.6945	219.6694	210.5889
Desvenlafaxine	193.2±21.8	204.2436	208.4600	180.0909	187.1062	234.4573
Diazepam	254.6±28.7	207.4026	209.3910	205.4755	208.7382	214.0107
Fluoxetine	192.8±27.9	232.0896	230.6235	216.9205	201.1350	250.3412
Imipramine	179.7±16.3	216.5286	216.0695	207.5810	226.1462	215.9245
Lorazepam	282.6±32.9	212.8431	212.1270	223.6108	215.0614	218.3004
Nortriptyline	194.9±15.0	209.3331	211.3385	200.6945	219.6694	210.5889
Oxazepam	266.2±32.9	207.4611	209.3815	205.0057	208.9430	213.9564
Protriptyline	198.3±12.3	209.3331	211.3385	200.6945	219.6694	210.5889
Trimipramine	183.3±16.3	222.2031	218.9290	235.2173	231.9574	222.0000

Table 4: Comparing the actual flash point with those determined computationally through regression modeling

**Name of the drug**	**Molar refractivity (cm^3)**	**Molar refractivity computed via the regression model with the Wiener Index**	**Molar refractivity computed via the regression model with the Hyper-Wiener Index**	**Molar refractivity computed via the regression model with the Harary Index**	**Molar refractivity computed via the regression model with the Detour Index**	**Molar refractivity computed via the regression model with the Detour Harary Index**
Alprazolam	88.2±0.5	86.2399	85.5067	91.3674	91.3465	85.1945
Amitriptyline	91.5±0.3	85.6459	85.5277	84.0661	88.5481	85.7413
Amoxapine	86.8±0.5	86.3749	85.5970	90.9266	93.0425	84.7123
Buspirone	106.8±0.4	107.6779	107.0485	105.0543	101.0879	94.1719
Clomipramine	93.8±0.3	87.1714	86.3971	89.1618	91.5161	86.4089
Desipramine	84.2±0.3	83.9854	84.4819	82.7294	85.8663	84.7579
Desvenlafaxine	77.8±0.3	82.8109	83.8456	78.7296	72.3831	89.1574
Diazepam	80.9±0.5	83.5399	84.0514	83.6575	81.3401	85.3886
Fluoxetine	79.9±0.3	89.2369	88.7449	85.8793	78.1919	92.0851
Imipramine	88.9±0.3	85.6459	85.5277	84.0661	88.5481	85.7413
Lorazepam	81.0±0.5	84.7954	84.6562	87.1781	81.4249	86.1793
Nortriptyline	86.8±0.3	83.9854	84.4819	82.7294	85.8663	84.7579
Oxazepam	76.4±0.5	83.5534	84.0430	83.5663	81.4249	85.3786
Protriptyline	84.8±0.3	83.9854	84.4819	82.7294	85.8663	84.7579
Trimipramine	93.5±0.3	86.9554	86.1598	89.4313	90.9543	86.8612

Table 5: Comparing the actual molar refractivity with those determined computationally through regression modeling

**Name of the drug**	**Polarizability** (10^{-24} cm^3)	**Polarizability computed via the regression model with the Wiener Index**	**Polarizability computed via the regression model with the Hyper-Wiener Index**	**Polarizability computed via the regression model with the Harary Index**	**Polarizability computed via the regression model with the Detour Index**	**Polarizability computed via the regression model with the Detour Harary Index**
Alprazolam	35.0±0.5	34.2308	33.8154	36.1807	36.2229	33.7888
Amitriptyline	36.3±0.5	33.9932	33.8234	33.2788	35.1141	34.0085
Amoxapine	34.4±0.5	34.2848	33.8498	36.0055	36.8949	33.5951
Buspirone	42.4±0.5	42.8060	42.0218	41.6206	40.0827	37.3944
Clomipramine	37.2±0.5	34.6034	34.1546	35.3041	36.2901	34.2765
Desipramine	33.4±0.5	33.3290	33.4250	32.7475	34.0515	33.6135
Desvenlafaxine	30.9±0.5	32.8592	33.1826	31.1578	28.7091	35.3804
Diazepam	32.1±0.5	33.1508	33.2610	33.1164	32.2581	33.8668
Fluoxetine	31.7±0.5	35.4296	35.0490	33.9994	31.0107	36.5563
Imipramine	35.3±0.5	33.9932	33.8234	36.1807	35.1141	34.1843
Lorazepam	32.1±0.5	33.6530	33.4914	34.5156	33.2955	33.8628
Nortriptyline	34.4±0.5	33.3290	33.4250	32.7475	34.0515	33.6135
Oxazepam	30.3±0.5	33.1562	33.2602	33.0801	32.2917	33.4250
Protriptyline	33.6±0.5	33.3290	33.4250	32.7475	34.0515	33.4275
Trimipramine	37.1±0.5	34.5170	34.0642	35.4112	36.0675	34.4582

Table 6: Comparing the actual polarizability with those determined computationally through regression modeling

**Name of the drug**	**Surface tension (dyne/cm)**	**Surface tension computed via the regression model with the Wiener Index**	**Surface tension computed via the regression model with the Hyper-Wiener Index**	**Surface tension computed via the regression model with the Harary Index**	**Surface tension computed via the regression model with the Detour Index**	**Surface tension computed via the regression model with the Detour Harary Index**
Alprazolam	52.2±7.0	46.4404	45.8771	49.7298	48.5285	46.1428
Amitriptyline	47.0±3.0	46.1148	45.8891	44.9583	47.4197	46.3430
Amoxapine	52.1±7.0	46.5144	45.9287	49.4417	49.2005	45.9662
Buspirone	62.4±5.0	58.1916	58.1867	58.6744	52.3883	49.4302
Clomipramine	41.7±3.0	46.9510	46.3859	48.2884	48.5957	46.5875
Desipramine	40±3.0	45.2046	45.2915	44.0848	46.3571	45.9829
Desvenlafaxine	47.9±3.0	44.5608	45.0455	41.4709	41.0147	47.5939
Diazepam	46.1±7.0	44.9604	45.0455	44.6913	44.5637	46.2139
Fluoxetine	33.0±3.0	48.0832	47.7275	46.1433	43.3163	48.6661
Imipramine	40.1±3.0	46.1148	45.8891	44.9583	47.4197	46.3430
Lorazepam	56.0±7.0	45.6387	45.0455	47.0128	45.7149	46.5034
Nortriptyline	47.3±3.0	45.2046	45.2915	44.0848	46.3571	45.9829
Oxazepam	54.6±7.0	44.9579	45.0455	44.6053	46.2814	46.2102
Protriptyline	41.2±3.0	45.2046	45.2915	44.0848	46.3571	45.9829
Trimipramine	39.1±3.0	46.8326	46.2503	48.4645	48.3731	46.7531

Table 7: Comparing the actual surface tension with those determined computationally through regression modeling

**Name of the drug**	**Molar volume (cm^3)**	**Molar volume computed via the regression model with the Wiener Index**	**Molar volume computed via the regression model with the Hyper-Wiener Index**	**Molar volume computed via the regression model with the Harary Index**	**Molar volume computed via the regression model with the Detour Index**	**Molar volume computed via the regression model with the Detour Harary Index**
Alprazolam	225.6±7.0	248.5084	246.3700	261.5126	261.1284	241.1551
Amitriptyline	257.8±3.0	246.5592	246.4410	243.7597	254.6868	244.3594
Amoxapine	228.2±7.0	248.9514	246.6753	260.4408	265.0324	238.3299
Buspirone	310.7±5.0	318.8568	319.2018	294.7921	283.5520	293.7542
Clomipramine	281.2±3.0	251.5651	249.3804	256.1498	261.5188	248.2704
Desipramine	254.3±3.0	241.1103	242.9052	240.5094	248.5136	238.5972
Desvenlafaxine	236.1±3.0	237.2562	240.7539	230.7841	217.4768	264.3738
Diazepam	225.9±7.0	239.6484	241.4497	242.7662	238.0948	242.2925
Fluoxetine	266.7±3.0	258.3430	257.3182	248.1685	230.8480	281.5276
Imipramine	269.2±3.0	246.5592	246.4410	243.7597	254.6868	244.3594
Lorazepam	211.2±7.0	243.7683	243.4945	251.3265	244.1216	246.9253
Nortriptyline	242.9±3.0	241.1103	242.9052	240.5094	238.2900	238.5972
Oxazepam	201.9±7.0	239.6927	241.4426	242.5444	238.0948	238.5972
Protriptyline	256.5±3.0	241.1103	242.9052	240.5094	248.5136	238.5972
Trimipramine	286.1±3.0	250.8563	248.5781	256.8051	260.2256	250.9206

Table 8: Comparing the actual molar volume with those determined computationally through regression modeling
